# Febuxostat Modulates MAPK/NF-*κ*Bp65/TNF-*α* Signaling in Cardiac Ischemia-Reperfusion Injury

**DOI:** 10.1155/2017/8095825

**Published:** 2017-08-24

**Authors:** Sana Irfan Khan, Rajiv Kumar Malhotra, Neha Rani, Anil Kumar Sahu, Ameesha Tomar, Shanky Garg, Tapas Chandra Nag, Ruma Ray, Shreesh Ojha, Dharamvir Singh Arya, Jagriti Bhatia

**Affiliations:** ^1^Department of Pharmacology, Cardiovascular Research Laboratory, All India Institute of Medical Sciences, New Delhi, India; ^2^Department of Anatomy, All India Institute of Medical Sciences, New Delhi, India; ^3^Department of Pathology, All India Institute of Medical Sciences, New Delhi, India; ^4^Department of Pharmacology and Therapeutics, College of Medicine and Health Sciences, United Arab Emirates University, Abu Dhabi, UAE

## Abstract

Xanthine oxidase and xanthine dehydrogenase have been implicated in producing myocardial damage following reperfusion of an occluded coronary artery. We investigated and compared the effect of febuxostat and allopurinol in an experimental model of ischemia-reperfusion (IR) injury with a focus on the signaling pathways involved. Male Wistar rats were orally administered vehicle (CMC) once daily (sham and IR + control), febuxostat (10 mg/kg/day; FEB10 + IR), or allopurinol (100 mg/kg/day; ALL100 + IR) for 14 days. On the 15th day, the IR-control and treatment groups were subjected to one-stage left anterior descending (LAD) coronary artery ligation for 45 minutes followed by a 60-minute reperfusion. Febuxostat and allopurinol pretreatment significantly improved cardiac function and maintained morphological alterations. They also attenuated oxidative stress and apoptosis by suppressing the expression of proapoptotic proteins (Bax and caspase-3), reducing TUNEL-positive cells, and increasing the level of antiapoptotic proteins (Bcl-2). The MAPK-based molecular mechanism revealed suppression of active JNK and p38 proteins concomitant with the rise in ERK1/ERK2, a prosurvival kinase. Additionally, a reduction in the level of inflammatory markers (TNF-*α*, IL-6, and NF-*κ*B) was also observed. The changes observed with febuxostat were remarkable in comparison with those observed with allopurinol. Febuxostat protects relatively better against IR injury than allopurinol by suppressing inflammation and apoptosis mediating the MAPK/NF-*κ*Bp65/TNF-*α* pathway.

## 1. Introduction

Ischemic heart disease is the major cause of morbidity and mortality worldwide. Ischemia stimulates metabolic and ionic disturbance in the myocardium and leads to rapid deterioration of cardiac function. Hence, thrombolytic therapy or primary percutaneous coronary intervention is imperative for salvaging the myocardium and improving clinical outcome [1]. However, restoration of blood flow to the ischemic myocardium termed reperfusion unavoidably leads to a number of complications such as stunning (diminished contractile function), rhythm abnormalities, and sequentially heart failure. Paradoxically, reperfusion may also induce death of cardiomyocytes which were viable prior to therapeutic procedure and can worsen infarct size, an entity termed myocardial ischemia-reperfusion (IR) injury [2]. Therefore, developments of effective therapeutic strategies are necessary to protect heart against the inimical consequences of reperfusion injury.

There are substantial evidences that oxidative stress plays a crucial role in IR injury [3]. Oxidative stress in turn produces mitochondrial impairment which leads to the release of inflammatory cytokines and subsequent cell death [4, 5]. Subsequently, there is stimulation of intracellular signaling pathways such as mitogen-activated protein kinases (MAPKs) [6]. The subfamilies of MAPKs identified in the cardiomyocyte are c-Jun NH-terminal kinases (JNKs), extracellular signal-regulated kinase-1/2 (ERK1/2, also known as p42/p44 MAPK), and p38 MAPKs [7]. The activation of MAPKs may further activate transcription factors, inducing exaggerated synthesis of proinflammatory cytokines, subsequently triggering inflammatory responses and institution of the apoptotic cascade [8]. NF-*κ*B is a nuclear transcription factor which regulates gene expression critical to inflammation and apoptosis during various pathologies including IR injury [9–11]. NF-*κ*B, when inactive, is sequestered in the cytoplasm, where it is bound by the I*κ*B family proteins including I*κ*B-*α*. Upon stimulation, NF-*κ*B gets phosphorylated and I*κ*B-*α* is degraded by IKK. The NF-*κ*B subunits translocate from the cytoplasm to the nucleus where they induce gene expression of inflammatory cytokines [11]. The role of NF-*κ*B subunit p65 has been demonstrated in different ischemic reperfusion injuries including cardiac injury [12–14].

Xanthine oxidase (XO) is involved in free radical generation following hypoxia as well as during reperfusion [15, 16]. Under normal physiological conditions, XO occurs mainly in the dehydrogenase form (XDH), while during reperfusion, the dehydrogenase form is converted to XO by posttranslational modifications [17]. Both XDH and XO are important enzymes that produce oxidative stress during reperfusion injury [18]. Allopurinol and its metabolites are structural analogues of both purines and pyrimidines, whereas febuxostat is a nonpurine inhibitor of XO. In contrast to allopurinol, febuxostat suppresses both oxidized and reduced forms of XO which may contribute to its superiority in limiting oxidative stress [17, 18]. Xanthine oxidase inhibitors (XOI) have demonstrated beneficial effects on myocardial IR injury [19]. The mitigation of oxidative stress, apoptosis, and inflammation by febuxostat has been extensively studied and documented [18, 19]. Febuxostat has also been shown to affect the MAPK pathway by inhibiting JNK phosphorylation in macrophages [20]. Since febuxostat has also demonstrated favorable outcomes in heart failure patients and ischemia-reperfusion injury is one of the precursors of heart failure, we proposed a hypothesis of the utility of febuxostat in reducing ischemia-reperfusion injury; thereby, febuxostat can be clinically translated as a preventive measure in heart failure. We have focused on the molecular pathways because to the best of our knowledge, there is no study depicting the role of XOI on MAPK modulation in the heart. Therefore, this study was conducted in order to investigate and delineate the downstream and upstream components of febuxostat- and allopurinol-mediated MAPK modulation in cardiac IR injury in rats.

## 2. Materials and Methods

### 2.1. Animals

Healthy adult male Wistar albino rats, 10 to 12 weeks old, weighing 150 to 200 g were used in the present study. The rats were maintained at standard laboratory conditions and provided food and water ad libitum. The experiments were conducted following the animal ethics approval from the Institutional Animal Ethics Committee of the All India Institute of Medical Sciences, New Delhi, India, with reference number IAEC No. 893/IAEC/15. The handling of the animals during experiments was carried out in accordance with the Indian National Science Academy Guidelines for the use and care of experimental animals.

### 2.2. Chemicals

Febuxostat (Febutaz) was purchased from Sun Pharma, Sikkim, India, and allopurinol (Zyloric) was purchased from GlaxoSmithKline, Mumbai, India. The drugs were suspended in carboxymethyl cellulose (0.5% CMC). The kits for TNF-*α* and IL-6 were purchased from RayBiotech Inc., Norcross, GA. The CK-MB kit was purchased from Logotech Private Limited, India.

The primary antibodies for caspase-3 (#9662S), Bcl-2 (#2876S), and *β*-actin (#4967S) were procured from Cell Signaling Technology, MA, USA, and those for Bax (#7480), NF-*κ*Bp65 (#109), p-NF-*κ*Bp65 (Ser536) (#101752), JNK (#572), p-JNK (#6254) (Thr 183/Tyr 185), ERK1/2 (#135900), p-ERK1/2 (#16982) (Thr 202/Tyr 204), and p-p38 (#17852) (Thr 180/Tyr 182) as well as anti-rabbit (#2004) and anti-goat (#2020) secondary antibodies were procured from Santa Cruz, California, USA. Antibody for p38 (#197348) was purchased from Abcam Technologies, USA.

### 2.3. Experimental Protocol

A total of 38 rats were randomly distributed into 4 groups. The doses of febuxostat and allopurinol were chosen based on a dose-response study in our laboratory [21] and previous studies [22]:

Group 1 (sham; *n* = 8): administered 0.5% CMC (2 mL/kg/day; p.o.) for 14 days. On day 15, IR surgery was performed and thread was passed beneath the left anterior descending (LAD) coronary artery but the coronary artery was not occluded.
Group 2 (IR-control; *n* = 10): administered 0.5% CMC (2 mL/kg/day; p.o.) for 14 days. On day 15, surgery was performed for LAD coronary artery ligation for 45 min following reperfusion for 60 min.
Group 3 (FEB10 + IR; *n* = 10): administered febuxostat (10 mg/kg/day; p.o.) for 14 days. On day 15, surgery was performed for LAD coronary artery ligation for 45 min following reperfusion for 60 min.
Group 4 (ALL100 + IR; *n* = 10): administered allopurinol (100 mg/kg/day; p.o.) for 14 days. On day 15, surgery was performed for LAD coronary artery ligation for 45 min following reperfusion for 60 min. There was no effect of febuxostat and allopurinol on normal heart (per se) in our preliminary morphological assessment (data not shown) which is in keeping with previous studies [19, 23].

### 2.4. Experimental Procedure for Myocardial Injury Induction and Hemodynamic Parameter Measurement

The experimental procedure followed for induction of ischemia and reperfusion and assessment of hemodynamic and ventricular function has been described by Agrawal et al. [24]. Briefly, following anesthesia with intraperitoneal injection of pentobarbitone sodium (60 mg/kg), monitoring and maintenance of body temperature were carried out at 37°C throughout the experiment. A ventral midline incision was made after opening the neck, a tracheostomy was performed, and the rats were ventilated with room air from a positive pressure respirator (TSE animal respirator, Germany) using compressed air at the rate of 70 strokes per minute and a tidal volume of 10 mL/kg. 0.9% saline was infused continuously through a polyethylene tube with which the left jugular vein was cannulated. After that, the right carotid artery was cannulated, and the cannula filled with heparinised saline was connected to a pressure transducer (Gould Statham P231D, USA) for the assessment of blood pressure (mean arterial pressure (MAP) and heart rate (HR)). A left thoracotomy was then performed at the fifth intercostal space, and the heart was exposed after opening the pericardium. After that, the LAD coronary artery was ligated using a 5-0 silk suture 4-5 mm from its origin with an atraumatic needle, and ends of this ligature were passed through a small vinyl tube to form a snare. Upon completion of this step, the heart was then returned back into the thorax. After stabilization of the animals for 15 minutes, LAD coronary artery ligation was carried out and myocardial ischemia was induced by one-stage occlusion of the LAD coronary artery by pressing the polyethylene tubing against the ventricular wall and then fixing it in place by clamping it with a hemostat (except in IR-sham rats). In addition to the above parameters, the maximum rates of rise and fall of left ventricular pressure (peak +LVdP/dt and peak −LVdP/dt) as well as of preload, that is, left ventricular end-diastolic pressure (LVEDP), were recorded using Biopac system software BSL 4.0 MP36 by introducing a sterile metal cannula into the cavity of the left ventricle. The ischemic region appeared grossly as an area of epicardial cyanosis. After 45 minutes, the snare was released gently thereby allowing reperfusion to commence which was evident from an area of hyperaemia in the previously cyanosed region. After 60 minutes of reperfusion, blood was drawn from the heart and rats were sacrificed with an overdose of pentobarbitone sodium (150 mg/kg; i.p.). Blood was withdrawn from the heart and centrifuged at 5000 rpm (Heraeus Biofuge, Germany) for 10 min to obtain serum for biochemical analysis. Hearts were then excised, rinsed in ice-cold saline, and stored for biochemical, histopathological, and ultrastructural evaluation; terminal deoxynucleotide transferase dUTP nick end labeling (TUNEL) assay; immunohistochemistry (IHC); and Western blot analysis. For biochemical estimations, hearts were stored in liquid nitrogen, whereas for immunohistochemistry, hearts were fixed in 10% buffer formalin.

### 2.5. Assessment of Biochemical Parameters

A 10% homogenate of the heart was prepared in ice-cold phosphate buffer (0.1 M, pH 7.4), and an aliquot was used to estimate thiobarbituric acid reactive substance (TBARS) and reduced glutathione (GSH). The supernatant obtained following the centrifugation of homogenate at 4930*g* for 15 minutes was used for the estimation of catalase and superoxide dismutase (SOD) activities. Serum samples were used for the estimation of lactate dehydrogenase (LDH) and creatine kinase-MB (CK-MB) isoenzyme activities and interleukin-6 (IL-6) and tumor necrosis factor-*α* (TNF-*α*) levels.

### 2.6. Measurement of MDA Level and Reduced GSH Content

Tissue MDA level was measured by the method published by Ohkawa et al. [25]. This method assesses the ability of MDA to react with thiobarbituric acid in acidic conditions leading to the development of pink color adducts whose absorbance was read at 532 nm.

Reduced GSH level was assessed by the method described by Moron et al. [26]. This method is based on the generation of yellow color when 5,5′-dithiobis(2-nitrobenzoic acid) (DTNB) is added to compounds containing sulfhydryl group like glutathione. The homogenate was centrifuged with equal parts of 10% tricarboxylic acid (TCA) at 5000 rpm for 10 min. The supernatant containing glutathione (which has a thiol (-SH) group) reacted with DTNB at pH 8.0 to produce a yellow-colored ion whose concentration was measured at 412 nm.

### 2.7. Measurement of Activities of Enzymes SOD and CAT

The activity of SOD was determined by the method described by Marklund S and Marklund G [27] measuring the extent of inhibition of pyrogallol autoxidation at pH 8.4. The activity of CAT was determined by the method described by Aebi measuring difference in H_2_O_2_ extinction per unit time [28].

### 2.8. CK-MB and LDH Enzyme Activities

The activities of myocardial enzymes, CK-MB isoenzyme and LDH, were determined in serum according to the manufacturer's instructions which have been extensively described previously [29].

### 2.9. Estimation of Serum TNF-*α* and IL-6 Levels

The levels of proinflammatory cytokines, TNF-*α* and IL-6, were estimated in serum following the manufacturer's instructions as previously described [29].

### 2.10. Histopathological Evaluation

The protocol followed for histopathological studies was described previously [30]. The formalin-fixed and paraffin-embedded heart tissues were cut to get cross sections of 5 *μ*m thick. The sections were stained with hematoxylin and eosin (H&E) and examined by a pathologist using a light microscope (DeWinter Technologies, Italy). The pathologist was unaware of the experimental groups and treatments. Three hearts from each group were examined for histological examination and graded for the severity of changes using a score on a scale of severe (+++), moderate (++), mild (+), and nil (−).

### 2.11. Ultrastructural Evaluation

The protocol followed for ultrastructural studies was described previously [30]. Karnovsky's fixed heart tissues were processed and embedded in araldite CY212 to cut thin sections (70–80 nm) using an ultracut microtome (Reichert, Australia) and stained with uranyl acetate and lead acetate. The visualization was performed using a transmission electron microscope (Morgagni 268D, Fei Company, the Netherlands) operated at 80 kV and evaluated by a cytologist masked to the experimental groups and treatments.

### 2.12. Immunostaining for Myocardial Apoptosis Markers

The protocol followed for immunostaining for detection of apoptosis markers in the heart was described previously [30]. The sections after deparaffinization were processed for immunostaining using primary rabbit monoclonal antibodies (mAb) against Bax, Bcl-2, and caspase-3 for 48 h followed by incubation with horse radish peroxidase- (HRP-) conjugated secondary antibodies. 3,3-Diaminobenzidine (DAB) was added for the initiation of colorimetric reaction and assessment under the light microscope (DeWinter Technologies, Italy) following the methods described [30].

### 2.13. Terminal Deoxynucleotidyl Transferase dUTP Nick End Labeling

The TUNEL assay for analysis of apoptosis in heart sections was performed using the ApoBrdU DNA fragmentation assay kit (Biovision, USA; #K403-50), and the steps for the assessment are described previously [9]. The sections were deparaffinized and processed to visualize the antigen-antibody interaction, and the counterstaining was then done with hematoxylin visualized under a light microscope. At least 5 fields in each slide were examined for any TUNEL-positive cells in each group by the pathologist unaware of the experimental groups and treatments.

### 2.14. Assessment by Western Blot

The heart tissue homogenate was prepared using RIPA buffer supplemented with protease inhibitor cocktail. The protein concentration was measured by the method described by Bradford [31]. The protocol followed for Western blotting has been described previously [32]. Briefly, each well was loaded with equal amounts of proteins (40 μg) in 10% sodium dodecyl sulfate polyacrylamide gel electrophoresis (SDS-PAGE) and then transferred to the nitrocellulose transfer membrane (BioTrace™ NT, Pall Corporation, USA) which was blocked for 1 h with 3% bovine serum albumin or 5% nonfat dried milk and incubated for 12 h at 4°C with primary antibodies. The primary antibodies used were against *β*-actin, total and phosphorylated IKK-*β*, total and phosphorylated NF-*κ*Bp65, total and phosphorylated p38, total and phosphorylated JNK, and total and phosphorylated ERK1/2 which were detected with HRP-conjugated anti-rabbit/anti-goat secondary antibodies. The antibody-antigen complexes were visualized using an enhanced chemiluminescence kit (Thermo Fischer Scientific Inc., USA) under the FluorChem M Protein Imaging System (ProteinSimple, USA) and were quantified using ImageJ software.

### 2.15. Statistical Analysis

The data were expressed as mean ± SEM. Statistical analysis was performed by ANOVA followed by the multiple comparison post hoc Bonferroni test which was done using GraphPad InStat 3 software (GraphPad Software Inc., San Diego, USA). The value of *p* < 0.05 was considered statistically significant.

## 3. Results

### 3.1. Mortality

During the course of this study, 4 out of 38 rats (translating to a mortality of 11%) died; one rat died in the IR-control group, two died in the FEB10 + IR group, and one died in ALL100 + IR group. The rats that died were not considered during statistical analysis, and the reason of the death was excessive bleeding during either carotid artery cannulation or coronary artery ligation.

### 3.2. XOI Improved Hemodynamic and Ventricular Dysfunction after IR

**Figure 1:**
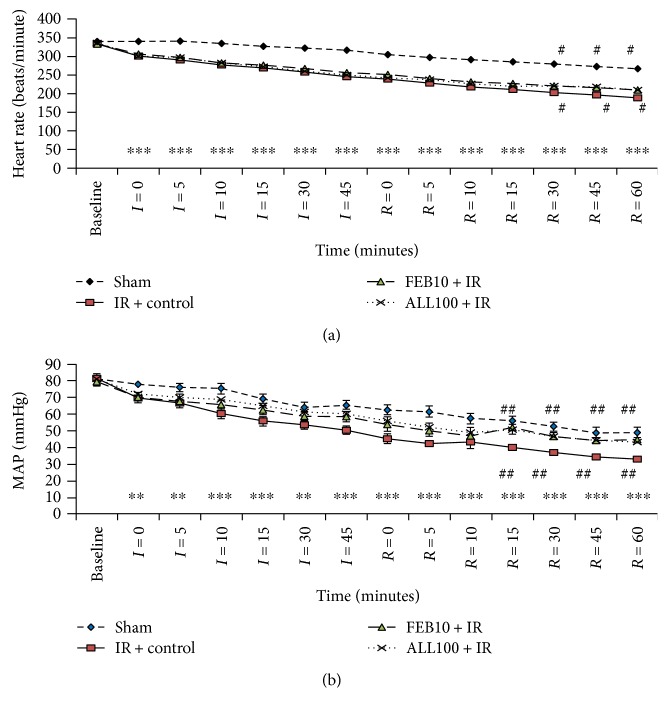
Effect of XOI on arterial pressure and heart rate. IR + control: ischemia-reperfusion control; FEB10 + IR: febuxostat 10 mg/kg/day + ischemia-reperfusion; ALL100 + IR: allopurinol 100 mg/kg/day + ischemia-reperfusion. Data are expressed as the mean ± SEM; *n* = 6 in each group. ^∗∗^*p* < 0.01 and ^∗∗∗^*p* < 0.001 versus sham; ^#^*p* < 0.05 and ^##^*p* < 0.01 versus IR + control.

**Figure 2:**
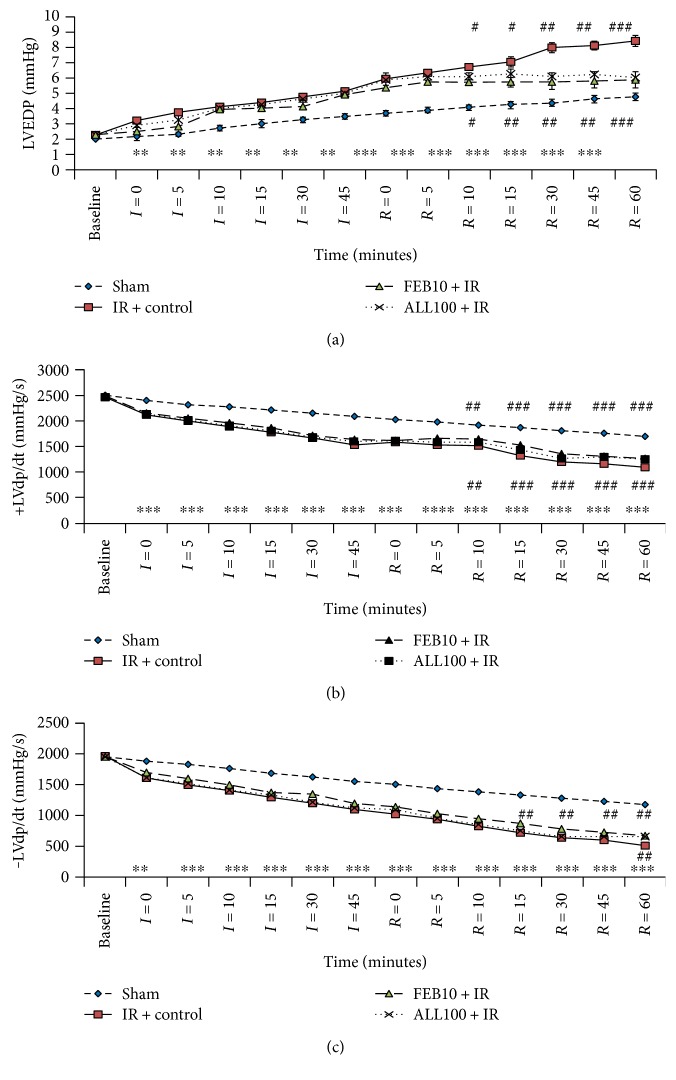
Effect of XOI on ventricular function. (a) LVEDP, (b) maximal positive rate of the left ventricular pressure (+LVdP/dtmax), and (c) maximal negative rate of the left ventricular pressure (−LVdP/dtmax). IR + control: ischemia-reperfusion control; FEB10 + IR: febuxostat 10 mg/kg/day + ischemia-reperfusion; ALL100 + IR: allopurinol 100 mg/kg/day + ischemia-reperfusion. Data are expressed as the mean ± SEM; *n* = 6 in each group. ^∗∗^*p* < 0.01 and ^∗∗∗^*p* < 0.001 versus sham; ^#^*p* < 0.05, ^##^*p* < 0.01, and ^###^*p* < 0.001 versus IR + control.

Reperfusion following cardiac ischemia in rats led to a significant fall in heart rate and mean arterial pressure (MAP) as compared to the sham group at all time points. Pretreatment with febuxostat (10 mg/kg) or allopurinol (100 mg/kg) significantly improved heart rate and MAP as compared to IR-control rats during reperfusion (Figure 1). The induction of IR also significantly depressed ventricular function as depicted by elevated LVEDP and reduced +LVdP/dtmax as well as −LVdP/dtmax as compared to the sham group at all time points (Figure 2). This was ameliorated by febuxostat and allopurinol during reperfusion. There was no significant difference between the improvements by allopurinol and febuxostat.

### 3.3. XOI Augmented Antioxidants, Improved Cardiac Injury, and Reduced Inflammatory Markers after IR

**Figure 3:**
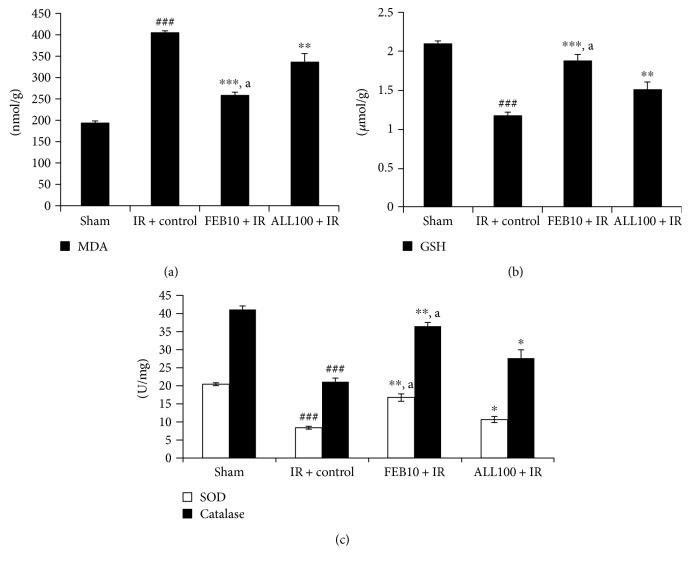
Effect of XOI on oxidative stress markers. (a) GSH; (b) MDA; (c) SOD and catalase. IR + control: ischemia-reperfusion control; FEB10 + IR: febuxostat 10 mg/kg/day + ischemia-reperfusion; ALL100 + IR: allopurinol 100 mg/kg/day + ischemia-reperfusion. Data are expressed as the mean ± SEM; *n* = 6 in each group. ^∗^*p* < 0.05, ^∗∗^*p* < 0.01, and ^∗∗∗^*p* < 0.001 versus IR + control; ^###^*p* < 0.001 versus sham. ^a^*p* < 0.05 versus ALL100 + IR.

As depicted in Figure 3, there was a significant (*p* < 0.001) rise in oxidative stress as evidenced by an increase in the lipid peroxidation product, MDA, with a significant (*p* < 0.001) fall in the antioxidant system, that is, GSH, SOD, and catalase levels in the IR-control group when compared to the sham group. Febuxostat and allopurinol pretreatment in IR-injured rats significantly (*p* < 0.001, *p* < 0.01) reduced the level of MDA concomitant with the significant (*p* < 0.001, *p* < 0.01) rise in GSH level and also increased the activities of the endogenous antioxidants SOD and catalase in comparison to the IR-control group. Improvement by febuxostat was more significant (*p* < 0.05) when compared to that by allopurinol.

**Figure 4:**
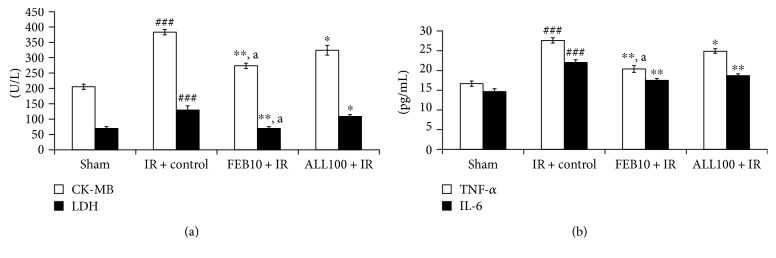
Effect of XOI on cardiac injury and inflammatory markers. (a) CK-MB and LDH; (b) TNF-*α* and IL-6. IR + control: ischemia-reperfusion control; FEB10 + IR: febuxostat 10 mg/kg/day + ischemia-reperfusion; ALL100 + IR: allopurinol 100 mg/kg/day + ischemia-reperfusion. Data are expressed as the mean ± SEM; *n* = 6 in each group. ^∗^*p* < 0.05 and ^∗∗^*p* < 0.01 versus IR + control; ^###^*p* < 0.001 versus sham. ^a^*p* < 0.05 versus ALL100 + IR.

As illustrated in Figure 4, there was a significant (*p* < 0.001) increase in the CK-MB isoenzyme and LDH in the IR-control group when compared to the sham group. The XOIs also significantly (*p* < 0.01, *p* < 0.05) preserved the level of the CK-MB isoenzyme and LDH, respectively. Improvement by febuxostat was more significant (*p* < 0.05) when compared to that by allopurinol. Since inflammation is a critical point in IR injury, we assessed TNF-*α* and IL-6 in the serum as these are usually low in normal heart. As anticipated, we observed a significant (*p* < 0.001) increase in cytokines TNF-*α* and IL-6 in the IR-control group when compared to the sham group, while febuxostat and allopurinol treatment significantly downregulated TNF-*α* (*p* < 0.001 and *p* < 0.05) and IL-6 (*p* < 0.001 and *p* < 0.05) (Figure 4). Improvement by febuxostat was more significant (*p* < 0.05) when compared to that by allopurinol with TNF-*α* but not with IL-6.

### 3.4. XOI Preserved Myocardial Architecture Histologically and Ultrastructurally after IR

**Figure 5:**
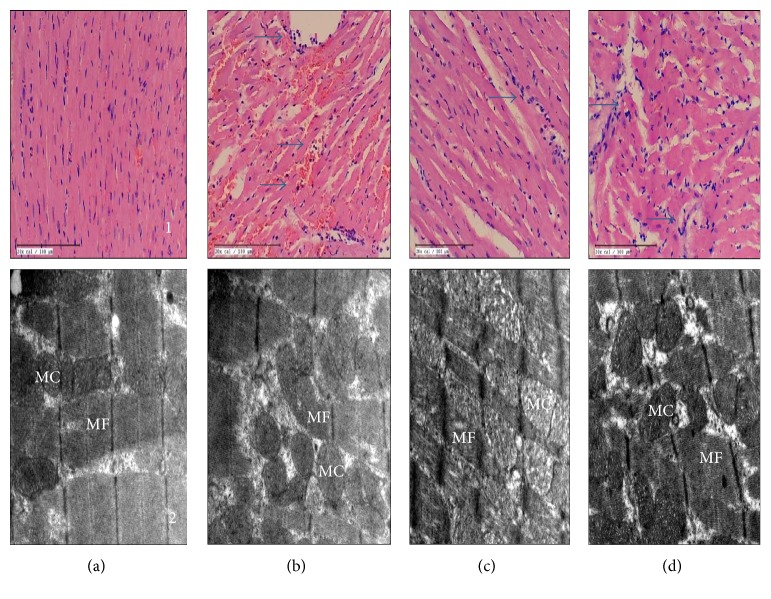
Effect of XOI on (a1–d1) histopathology (*n* = 3) (20x; scale bar 100 μm) and (a2–d2) ultrastructure (*n* = 3). (a) Sham; (b) IR + control: ischemia-reperfusion control; (c) FEB10 + IR: febuxostat 10 mg/kg/day + ischemia-reperfusion; (d) ALL100 + IR: allopurinol 100 mg/kg/day + ischemia-reperfusion.

The light microscopic examination of heart sections from the myocardial IR group showed myofibrillar membrane damage with extensive myonecrosis, edema, and inflammatory cell infiltration as compared to the light microscopic examination of heart sections from the sham group (Figure 5). On the other hand, XOI pretreatment in the IR rats showed scanty areas of myofibril loss with necrosis with little edema and inflammatory cell infiltration. However, the degree of myofibril loss, necrosis, edema, and inflammatory cell infiltration seen with allopurinol was higher than that seen with febuxostat (Table 1). Ultrastructural examination by electron microscopy revealed cardiomyocytes with intact myofibrils, mitochondria with well-preserved cristae, and nucleus with uniformly dispersed chromatin in the sham group (Figure 5). Loss of cytoplasmic organelles, myofibril disintegration, presence of lipid droplets, mitochondrial damage, and chromatin condensation were observed in the IR-control group. Pretreatment with febuxostat and allopurinol displayed lesser mitochondrial swelling and cristae disruption and fewer vacuoles with a better preservation seen with febuxostat as compared to allopurinol.

### 3.5. XOI Modulated Apoptosis, MAPKs, and Inflammation after IR

**Figure 7:**
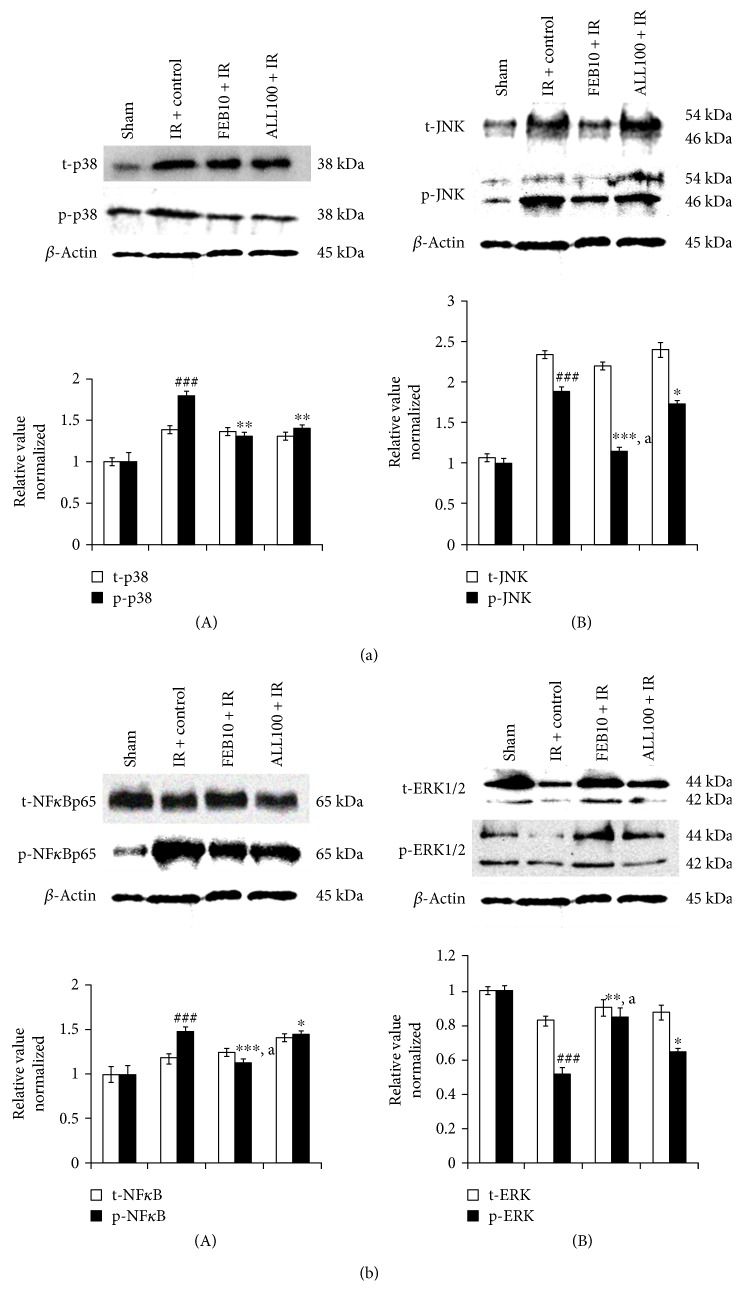
Effect of XOI on MAPK protein expressions. (a) (A) p38 and p-p38 and (B) JNK and p-JNK; (b) (A) NFκBp65 and p-NFκBp65 and (B) ERK1/2 and p-ERK1/2. Protein expressions are normalized with *β*-actin. All the values are expressed as mean ± SEM; *n* = 3 in each group. ^∗^*p* < 0.05, ^∗∗^*p* < 0.01, and ^∗∗∗^*p* < 0.001 versus IR + control; ^###^*p* < 0.001 versus sham. ^a^*p* < 0.05 versus ALL100 + IR.

The underlying molecular mechanism through which XOI exerts protection following cardiac IR was evaluated. The expression of various key regulatory proteins (caspase-3, Bax, and Bcl-2) in apoptosis was studied using immunohistochemistry studies. The TUNEL assay was also carried out to detect DNA fragmentation in apoptotic nuclei. Photomicrographs represented in Figure 5() demonstrate the effect of febuxostat and allopurinol on caspase-3, Bax, and Bcl-2 proteins and TUNEL-positive cells, respectively. As anticipated, expressions of caspase-3 and Bax proteins were increased and that of Bcl-2 was decreased in the IR-control group as compared to the sham group. Moreover, TUNEL-positive cells were increased in the IR-control rats as compared to the sham group (Figure 5()). The XOI pretreatment decreased caspase-3 and Bax and increased Bcl-2 levels. Further, it also reduced TUNEL-positive cells when compared with IR-control group. It was remarkable that the changes produced in the febuxostat group were more marked in comparison to those produced in the allopurinol group. We further demonstrated the role of inflammation in IR injury by assessing the expression of NF-κBp65 in the myocardium using Western blot. In rats subjected to IR, we observed significantly (*p* < 0.001) increased expression of NF-κBp65 in the myocardium, whereas XOI pretreatment significantly reduced its expression when compared to IR-control (Figure 7(b)). The levels of inflammatory markers were reduced more significantly by febuxostat (*p* < 0.05) as compared to allopurinol. We further assessed whether the cardioprotective effect of XOI was MAPK dependent by investigating their effect on phosphorylation of MAPK proteins. We did not observe any significant change in total p38, JNK, and ERK protein expressions in any of the groups. Though there was a significant (*p* < 0.001) decrease in expression of p-ERK and increase in expressions of p-p38 and p-JNK in the IR-control group as compared to the sham group. These findings were normalized by febuxostat (p-ERK: *p* < 0.01, p-JNK: *p* < 0.001, and p-p38: *p* < 0.001) and allopurinol (*p* < 0.05) pretreatment. Interestingly, febuxostat increased p-ERK and decreased p-p38 and JNK more significantly (*p* < 0.05) as compared to allopurinol (Figures 7(a) and 7(b)).

## 4. Discussion

Although numerous studies have described the fundamental role of XO in IR injury, however, the molecular mechanisms underlying the inhibition of XO in cardiac IR injury are not known. Thus, our study design attempted to examine the effects of selective and potent inhibitor of XO, febuxostat, in comparison to the classical XOI, allopurinol, in a rat model of myocardial infarction. In this study, we observed that febuxostat significantly diminished ischemia-reperfusion-induced myocardial injury and the effect was superior to that of allopurinol. This was evident from suppression of oxidative stress and apoptosis in the febuxostat treatment group. We further explored the effect of febuxostat pretreatment on MAPK-mediated inflammation and observed that febuxostat pretreatment ameliorated the cardiac dysfunction induced by reperfusion injury, primarily via the activation of the ERK1/2 pathway and suppression of the p38/JNK/NF-*κ*Bp65/TNF-*α* pathway. Further, febuxostat modulated these regulatory proteins more markedly than allopurinol.

The rat model of myocardial IR injury is a clinically relevant model and mimics the human pathophysiological condition. This IR model has been standardized as ischemia for 45 min followed by reperfusion for 60 min in our laboratory [9, 30, 33]. The occlusion of the LAD coronary artery results in myocardial ischemia which initiates complex cascade of cellular events leading to myocardial cell death. Reperfusion (restoration of blood flow), on the other hand, aggravates myocardial injury, induces metabolic derangement, and diminishes cardiac contractile function. In the present study, the IR group exhibited significant impairment of systolic and diastolic function, depicted by a decrease in MAP, as well as by impaired inotropic and lusitropic state, represented by a fall in +LVdP/dtmax and −LVdP/dtmax, respectively, along with increased LVEDP which characterizes increased preload on the compromised heart. These hemodynamic changes were improved by pretreatment with febuxostat or allopurinol. A more pronounced and earlier onset of improvement was observed in the febuxostat group. This improvement could be attributed to the faster acting property of febuxostat. Additionally, the histological examination of heart sections in the IR group revealed inflammatory cells, necrosis, edema, and replacement of myofibrils by interfibrillar spaces. The pretreatment with XOI mitigated these changes. Further, pretreatment with febuxostat showed more marked restoration as compared to that with allopurinol. In the IR group, ultrastructural findings further corroborated the myocardial injury and swollen as well as irregular mitochondria with loss of cristae and chromatin condensation, whereas pretreatment with febuxostat showed only mild separation of the mitochondrial cristae with negligible swelling and vacuolation. This cardioprotective effect was less pronounced in the allopurinol group. The plausible reason for the cardioprotective effect of febuxostat is the inhibition of XO-mediated oxidative stress and inflammation and augmented mitochondrial protection.

XO-mediated oxidative stress has been extensively demonstrated by numerous investigators [1, 15, 33]. Its levels have also been found to increase in heart tissue during reperfusion following myocardial ischemia [15, 17]. Ischemic injury leads to energy charge depletion from the cell that subsequently results in accumulation of hypoxanthine from ATP breakdown as well as concomitant conversion of xanthine dehydrogenase (XDH) to its XO isoform, a reaction which is mediated by sulfhydryl oxidation [17]. XDH exhibits NADH oxidase activity under acidic conditions like ischemia and XDH oxidizes NADH rather than xanthine, it has also been shown that a tissue likely remains in a reductive state (low NAD+-to-NADH ratio) in the early reperfusion period which favors oxidative stress in terms of ROS generation by XDH. Therefore, accumulated hypoxanthine as well as a reductive state (higher NADH relative to NAD+) during reperfusion produces a burst in superoxide production [17, 34]. Interestingly, while allopurinol can inhibit the generation of superoxide by XO, the drug has no effect on the NADH oxidase activity of XDH. Therefore, we hypothesized that febuxostat should be superior to allopurinol in ameliorating myocardial IR injury. Febuxostat has been shown to attenuate pressure overload in the left ventricle as well as it protects the kidneys from IR injury via suppression of oxidative stress [19, 35]. From our study, it is evident that febuxostat alleviated oxidative stress by restoring the antioxidant enzymes SOD and catalase and preventing depletion of GSH concomitant with the inhibition of lipid peroxidation as evidenced by decreased MDA formation. Febuxostat exerted a significantly better antioxidant effect than allopurinol. Oxidative stress-mediated lipid peroxidation also denatures cellular proteins and DNA resulting in loss of membrane integrity. Consequently, cardiac enzymes such as LDH and CK-MB are released from the intracellular compartment to the extracellular fluid [36, 37]. In our study, we observed that cardiac enzyme levels were raised in the IR-control group. However, febuxostat pretreatment reduced the serum LDH and CK-MB levels more potently as compared to allopurinol pretreatment. This further validates that febuxostat has a superior antioxidant property. Studies have proposed that MAPK pathway activation is mediated via oxidative stress by the inactivation of MAPK phosphatases (MKPs). It has been demonstrated that ERK is responsible for promoting cell survival, while p38 MAPKs/JNKs are involved in cell death and a tight regulation of these pathways is an important determinant of cell survival [37–39].

The free radicals generated from the mitochondria induces DNA damage and membrane peroxidation that promotes outer membrane permeabilization and facilitates translocation of Bax and cytochrome C from the mitochondria to the cytosol with subsequent activation of caspases to initiate and execute apoptosis [32, 40]. The antiapoptotic protein, Bcl-2, maintains the external mitochondrial membrane integrity and thereby prevents the release of cytochrome C from the mitochondria. On the contrary, the proapoptotic protein, Bax, induces mitochondrial injury that results in cell death [41, 42]. MAPK also arbitrates apoptosis directly by activating Bax during reperfusion. The apoptosis-induced cardiac damage following IR is halted by the activation of ERK1/ERK2 [43]. There is substantial evidence regarding improvement of cardiac function following IR injury by suppressing cardiomyocyte apoptosis through targeted inhibition of p38 MAPK [36, 39, 44]. The present study findings reveal for the first time that febuxostat modulates the MAPK in the heart tissue as evidenced by enhanced activities of ERK1/ERK2, increased Bcl-2, decreased Bax, reduced caspase-3, and fewer DNA fragments in febuxostat-treated groups relative to the untreated group. This observation is in affirmation with the MAPK-regulating property of febuxostat explored previously in the macrophages [20], whereas allopurinol, on the other hand, demonstrated a milder effect when compared to febuxostat. It appears from the present study that MAPK is a downstream component of oxidative stress and an upstream component of apoptosis. Furthermore, we can convincingly state that oxidative stress can activate apoptosis directly as well as indirectly by modulation of the MAPK pathway.

The components of the MAPK pathway are known to induce nuclear factor kappa-light-chain-enhancer of activated B cells (NF-*κ*B), an important inflammatory transcription factor [10, 17, 34]. NF-*κ*B plays a key role in the process of inflammation and apoptosis induced by IR injury [10]. Previous studies have shown that the NF-*κ*B subunit p65 is activated during IR injury in the rat steatotic liver [13]. This has been further supported by the suppression of NF-*κ*B using its inhibitor BAY 11-7802 or the inhibition of I*κ*B phosphorylation which has been shown to reduce the inflammation and apoptosis induced by myocardial as well as cerebral IR injury [12, 14]. Previous studies have also shown that NF-*κ*B activation is associated with the release of proinflammatory cytokines such as TNF-*α* [9, 13, 18]. In addition, the TNF-*α*-induced secretion of IL-6 is mediated by NF-*κ*B signaling as evidenced by reduced expression of IL-6 when NF-*κ*B is inhibited [45]. It was also evident from our study that upon reperfusion, there was increased phosphorylation of NF-*κ*B and I*κ*B, along with increased expression of TNF-*α* and IL-6. This transcription factor phosphorylation and cytokine increase were significantly diminished by febuxostat pretreatment. This is in agreement with the previously documented NF-*κ*B inhibitory effect of febuxostat [34]. In the present study, however, a significantly lower protection was observed with allopurinol. These two drugs have been previously compared in intestinal IR injury where febuxostat emerged superior to allopurinol in ameliorating the reperfusion injury [22]. Wang and colleagues have also demonstrated the antioxidant and antiapoptotic effect of XOI on cardiac ischemia-reperfusion injury *in vitro* and *in vivo* [19]. To our knowledge, the present study for the first time focused on the signaling pathway through which XOI mediated their cardioprotective effect. A convincing reason for the superior effect of febuxostat as a cardioprotective agent in IR injury is the ability of this drug to inhibit both XDH and XO thereby abolishing the NADH-derived as well as hypoxanthine-derived sources of free radical-mediated cell damage.

To conclude, using an experimental model of cardiac IR injury, our study provides convincing data on the improved efficacy of febuxostat in ameliorating the cardiac damage induced by IR injury following LAD coronary artery occlusion. The effect of febuxostat appears to be mediated by attenuation of XO-mediated oxidative stress as well as suppression of apoptosis and inflammation mediating the MAPK signaling pathway. In view of the favorable properties of febuxostat vis a vis allopurinol, the former may be further evaluated for its usefulness in halting the damage produced in the heart by revascularization procedures in humans.

## Figures and Tables

**Figure 6:**
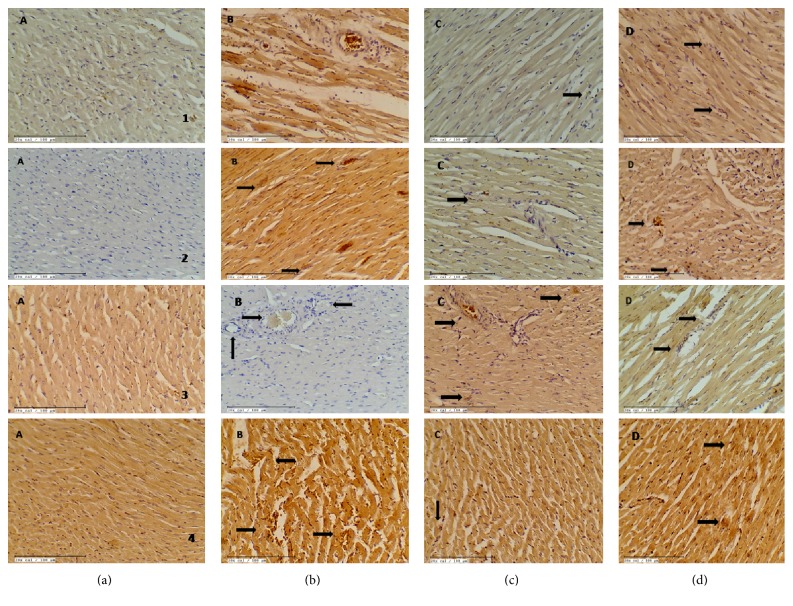
Effect of XOI on (a1–d1) caspase-3 immunohistochemistry (*n* = 3) (20x; scale bar 100 μm), (a2–d2) Bax immunohistochemistry (*n* = 3) (20x; scale bar 100 μm), (a3–d3) Bcl-2 immunohistochemistry (*n* = 3) (20x; scale bar 100 μm), and (a4–d4) TUNEL positivity (20x; scale bar 100 μm). (a) Sham; (b) IR + control: ischemia-reperfusion control; (c) FEB10 + IR: febuxostat 10 mg/kg/day + ischemia-reperfusion; (d) ALL100 + IR: allopurinol 100 mg/kg/day + ischemia-reperfusion.

**Table 1:** Effect of XOI on histological scoring of cardiac tissue.

	Myonecrosis	Inflammatory cells	Edema
Sham	neg	neg	neg
IR-C	3+	3+	4+
FEB10 + IR	1+	1+	1+
ALL100 + IR	2+	2+	2+

(3+) severe; (2+) moderate; (+) mild; (neg) nil. IR-C: ischemia-reperfusion control; FEB10 + IR: febuxostat 10 mg/kg/day + ischemia-reperfusion; ALL100 + IR: allopurinol 100 mg/kg/day + ischemia-reperfusion.
